# Survival and prognostic factors for relapsed childhood acute lymphoblastic leukemia after treatment with the Chinese children’s cancer group ALL-2015 protocol: a single center results

**DOI:** 10.3389/fonc.2024.1405347

**Published:** 2024-10-11

**Authors:** Xia Chen, Jie Yu

**Affiliations:** Department of Hematology and Oncology, Children’s Hospital of Chongqing Medical University, National Clinical Research Center for Child Health and Disorders, Ministry of Education Key Laboratory of Child Development and Disorders, Chongqing Key Laboratory of Pediatric Metabolism and Inflammatory Diseases, Chongqing, China

**Keywords:** childhood acute lymphoblastic leukemia, relapse, Chinese children’s cancer group ALL-2015 protocol, survival, prognostic factors

## Abstract

**Introduction:**

This retrospective study was conducted to assess the survival rates and prognostic factors in children with relapsed acute lymphoblastic leukemia (ALL) who were treated according to the Chinese Children’s Cancer Group ALL-2015 protocol at the Children’s Hospital of Chongqing Medical University.

**Methods:**

The study cohort involving 852 evaluable children with ALL reported a total of 146 relapses during a median follow-up period of 53 months. The primary outcomes measured were the second complete remission (CR2) rates, and 5-year event-free survival (EFS) and overall survival (OS) for patients who received re-treatment post-relapse. Patient data were stratified by ALL subtype (B-ALL vs. T-ALL), age at relapse, site of relapse, and timing of relapse. Univariate and multivariate analyses were performed to identify factors significantly associated with EFS and OS.

**Results:**

As of March 31, 2023, 146 relapses were observed, including 128 B-ALL and 18 T-ALL cases. The 8-year CIR was (19.8 ± 1.6)%, with no significant difference between B-ALL and T-ALL (P=0.271). Among the 105 patients who underwent re-treatment, 70 achieved CR2, resulting in a CR2 rate of 67.6%. The 5-year EFS and OS rates for re-treated patients were (45.0 ± 5.4)% and (56.9 ± 5.2)%, respectively. Significant differences in 5-year OS and EFS were found between B-ALL and T-ALL relapses (P < 0.001). The 5-year EFS and OS varied significantly with relapse timing and site of relapse. Factors significantly affecting EFS after relapse included the site of relapse, immunophenotyping, CR2 achievement, and hematopoietic stem cell transplantation (HSCT). Immunophenotyping, CR2 achievement, and HSCT were also identified as significant factors affecting OS after relapse.

**Discussion:**

Despite treatment with the CCCG-ALL-2015 protocol, a significant relapse rate was observed, with 72% of children opting for re-treatment post-relapse. The study highlights the importance of considering specific prognostic factors to inform tailored treatment strategies for relapsed childhood ALL. The findings emphasize the need for further research into improving therapeutic approaches for this patient population. This retrospective study was conducted to assess the survival rates and prognostic factors in children with relapsed acute lymphoblastic leukemia (ALL) who were treated according to the Chinese Children’s Cancer Group ALL-2015 protocol at the Children’s Hospital of Chongqing Medical University.

## Introduction

Presently, the cure rate for acute lymphoblastic leukemia (ALL) in children has surpassed 90% through the application of global protocols established by multi-center collaborative groups such as COG and BFM. However, despite these advancements, relapse remains a significant contributor to treatment failure in pediatric ALL, occurring in approximately 10% to 20% of children undergoing chemotherapy, with survival rates post-relapse hovering around 50% (1, 2). Numerous extensive studies have been undertaken to confront this formidable disease. The BFM of Relapsed Acute Lymphoblastic Leukemia Study Group, established since the 1990s, has identified critical prognostic factors, including time of relapse, site of relapse, and immunophenotyping. The prognosis for relapsed T-ALL is bleak, with less than 10% long-term survival for T-ALL bone marrow relapse post-chemotherapy, necessitating the recommendation of allogeneic hematopoietic stem cell transplantation (HSCT) (3, 4). Drawing from prior research, patients were classified into standard, intermediate, and high-risk categories based on the site of relapse, time of relapse, and immunophenotyping. This stratification approach, shared by BFM, UK, and COG, is consistent across various studies and serves as the basis for treatment risk groupings. Our center adopted the CCCG-ALL-2015 protocol for newly diagnosed ALL children. Treatment regimens for relapse encompassed the modified relapsed ALL2017 protocol, drawing from the UK2003 protocol, the Hong Kong 2007 protocol, and the chemotherapy protocol for continuing the initial diagnosis. This study delves into the clinical data of children treated with the CCCG-ALL-2015 protocol who experienced relapse from January 2015 to December 2019. The objective is to comprehend the survival outcomes of children with ALL after relapse and investigate the factors influencing survival prognosis after relapse. Notably, this marks the first clinical study in China reporting on the survival of children with ALL after relapse, treated with the CCCG-ALL-2015 protocol, and explores the factors impacting survival prognosis after relapse.

## Patients and methods

### Participants

The study involved 146 children who experienced a relapse and were treated with the CCCG-ALL-2015 protocol at the Children’s Hospital of Chongqing Medical University between January 2015 and December 2019. Inclusion criteria for participants were as follows: (1) children aged less than 18 years with the first diagnosis of ALL, (2) previous chemotherapy administered according to the CCCG-ALL-2015 protocol before relapse, and regular follow-up, and (3) relapse diagnosed based on clinical manifestations and corresponding laboratory tests. The study received approval and adoption from the Ethics Committee of Children’s Hospital of Chongqing Medical University.

### Treatment protocol

The primary diagnosis of ALL was managed with the CCCG-ALL-2015 protocol, which involved risk-based stratification for chemotherapy (**Table 1**). This encompassed distinct periods of treatment, including the induction remission period, consolidation treatment period, and continuation treatment period. In the event of relapse, chemotherapy regimens were adapted, primarily incorporating the Hong Kong 2007 protocol, the relapsed ALL 2017 protocol, and the continuation of the initial chemotherapy regimen (**Table 2**). Regular monitoring of Minimal Residual Disease (MRD) was conducted using flow cytometry. MRD monitoring is crucial for assessing the effectiveness of treatment and identifying any residual leukemia cells that may not be detectable through standard diagnostic methods. At the time of initial treatment, MRD was detected on the 19th day of induced remission and at the end of induced remission (46th day). After relapse, MRD was detected after the second induced remission. This comprehensive approach aimed to not only address the relapse but also tailor the subsequent treatment based on the specific circumstances of each patient. The combination of the CCCG-ALL-2015 protocol and the adapted regimens after relapse reflects a multidimensional strategy to manage pediatric ALL, considering risk factors, response to treatment, and disease characteristics.

**Table 1:** The risk grouping.

Low-risk ALL	1)aged between 1 year and <10 years; 2)Patients with B-cell ALL; 3)leukocyte count <50 × 109/L; 4)hyperdiploidy >50 chromosomes; 5)ETV6-RUNX1 oncogene fusion and without CNS3 status; 6)testicular leukemia; 7)MRD <1% on Day 19 of induction, and MRD <0.01% on Day 46 of induction were classified as having low-risk disease.
High-risk ALL	MRD ≥1% in bone marrow on Day 46 of induction and infants younger than 6 months with KMT2A rearrangement and leukocyte count ≥300 × 10^9^/L were considered to have high-risk ALL.
Intermediate-risk ALL	The remaining cases were classified as intermediate-risk ALL

**Table 2:** The CCCG-ALL-2015 protocol and relapsed ALL 2017 protocol.

	CCCG-ALL-2015 protocol	relapsed ALL 2017 protocol
remission induction	dexamethasone for 4–5 days as upfront window therapy; VDLP:prednisone, vincristine, daunorubicin, and pegaspargase from Day 5 to Day 28; CAT:cyclophosphamide, mercaptopurine, and cytarabine from Day 29 to Day 35; CAT+:MRD ≥1% on Day 19 of remission induction and IR/HR, T-ALL; IT:1)LR: d5, d19, d29; 2)IR: d5, d12, d19, d29; 3)HR: d5, d8, d12, d15, d19;	4 weeks(week1-4) Dex 20 mg/m^2^,D1-5, 15-19; VCR1.5mg/m^2^,D3, 10, 17, 24; Mitoxantrone 10mg/m^2^,D1, 2; Peg Asp 1000U/m^2^,D3, 17; IT: D1, D8;
consolidation	6-MP 25 mg/m^2^/d d1-56; HD-MTX: LR:3g/m^2^*4, d1、15、 29、 43; IR/HR:5 g/m^2^*4, d1、d15、 d29、 d43; IT: d1、d15、 d29、 d43;	4 weeks (week 5-8) Dex 6mg/m^2^, D1-5; VCR 1.5 mg/m^2^, D3; MTX 1-5g/m^2^, D8; Peg Asp 2000u/m^2^, D9; CTX 440 mg/m^2^, D15-19; Etoposide 100 mg/m^2^, D15-19 IT:D8; 4 weeks (week 9-13) Dex 6mg/m^2^, D1-5; VCR1.5mg/m^2^, D3; Cytarabine 3gm Q12H D1, 2, 8, 9; Erwinase 20000U/m^2^, D2, 4, 9, 11, 23; MTX 1-5gm/m^2^, D22; IT: D1, 22;
continuation	Phase 1: Interval and reinduction therapy Low risk(LR): 6-MP+VCR+Dex; Dex+VCR+DNR+L-Asp Intermediate risk/High risk(IR/HR): 6-MP+VCR+Dex+PEG-Asp+DNR; Dex+VCR+Ara-C+PEG-Asp; IT: LR: Day 1 of Weeks 1, 4, 7 and 13; IR/HR: Day 1 of Weeks 1, 4, 7 10, and 13; Phase 2: Maintenance therapy LR:6-MP+MTX/VCR/Dex; IR/HR:6-MP+MTX/CTX/Ara-C/VCR/Dex; 6-MP 50 mg/m2/d, qn;MTX:25 mg/m2,d8; IT: LR: Day 1 of week 4; IR/HR: Day 1 of week 3; The patients with IR/HR ALL received 6 weeks of mercaptopurine and methotrexate, followed by 1 week of treatment with cyclophosphamide. The total course of treatment is about 2.5 years.	Continuation(week14-21,22-29) Dex 6 mg/m^2^, D1-5; VCR 1.5 mg/m^2^, D1; 6MP 75 mg/m^2^, D1-42; MTX 20 mg/m^2^, D8, 15, 29, 36; CTX 300 mg/m^2^, D43, 50; Etoposide 150 mg/m^2^, D43, 50; Thioguanine 40 mg/m^2^, D43-49; Cytarabine 50 mg/m^2^, D44-47, 51-54; IT D1, 43; Maintenance weeks cycle up to 104 weeks from start of Continuation (7.5 cycle) Dexa 6 mg/m^2^, D1-5, 29-33, 57-61; VCR 1.5 mg/m^2^,D1,29,57; 6MP 75mg/m^2^,daily; MTX 20 mg/m^2^, D8, 15, 22, 29, 36, 43, 50, 57, 64, 71, 78; IT: d1;

### Relevant definition

Relapse is defined as the reappearance of leukemia cells in peripheral blood or bone marrow with ≥20% blast cells after achieving complete remission, or the infiltration of leukemia cells outside the bone marrow (**Table 3**) (5).

**Table 3:** Definition of central nervous system state and relapse category.

Central nervous system status	
CNS1	No leukemic cells in cerebrospinal fluid and no abnormal clinical manifestations and imaging evidence.
CNS2	WBC in cerebrospinal fluid was less than 5/UL
CNS3	WBC of cerebrospinal fluid is > 5/UL and immature cell infiltration.
Site of relapse	
Isolated Bone Marrow Relapse	Defined as ≥20% blast cells in the bone marrow at any point after achieving remission, excluding invasion of the central nervous system (CNS), testicular, or other extramedullary sites.
CNS Relapse	Defined as positive cell morphology in cerebrospinal fluid and CNS3, or the presence of definite CNS involvement symptoms, signs, or imaging findings such as cranial nerve paralysis.
Testicular Relapse	Confirmed by leukemic invasion ascertained through ultrasound and confirmed by testicular biopsy once testicular enlargement is detected.
Isolated Extramedullary Relapse*	Characterized by leukemic cell infiltration in extramedullary tissues after complete remission. This includes instances of CNS relapse and testicular relapse.
Time of relapse	
very early relapse	within 18 months of initial diagnosis
early relapse	≥18 months but <36 months after initial diagnosis)
late relapse	≥36 months after initial diagnosis

*Combined relapse: bone marrow relapse with extramedullary invasion at any point after remission.

### Statistical analysis

Counting data were expressed as rates, and measurement data were expressed as mean ± standard deviation and interquartile interval. Chi-square tests were employed for comparing counting data, and t-tests were used for comparing measurement data between groups. Performed using the Kaplan-Meier method, survival curves were plotted to visualize the survival outcomes. The LogRank test was then applied to compare survival between different groups. All statistical results were considered statistically significant when the p-value was less than 0.05 (*P*<0.05).

## Results

### Treatment after relapse

A total of 146 relapses were observed among 852 evaluated children, with an 8-year CIR at (19.8 ± 1.6) %. Among the 146 relapsed children, very early simple bone marrow relapse was the most common (**Table 4**). 128 cases were classified as B-ALL, and 18 cases were identified as T-ALL. Among the 146 relapsed patients, 105 continued to receive chemotherapy and 41 abandoned for various reasons. Specifically, 85 adhered to the relapsed ALL 2017 protocol based on the UK2003 protocol, 15 followed the relapsed Hong Kong 2007 protocol, 5 patients with CNS leukemia continued to increase the number of sheath injections per the initial protocol, and 1 patient received FLAG protocol due to M5 after relapse. Thirty children underwent hematopoietic stem cell transplantation after achieving remission, and 5 children received CAR-T therapy. As of March 31, 2023, 52 patients succumbed to the disease after discontinuing treatment, while 71 patients achieved a second remission. The second complete remission (CR2) rate was 67.6%. Ten patients experienced a second relapse after achieving CR2, with the majority of these relapses occurring in very early or early bone marrow relapses (70%). Among these second relapse cases, 5 (50%) resulted in death due to the abandonment of treatment. Two cases underwent CAR-T therapy after a secondary relapse, and one case experienced a subsequent relapse even after CAR-T therapy (triple relapse).The initial risk for this child was categorized as low-risk, but the risk escalated to high-risk due to D19 and D46 MRD positivity following the initial induction of remission therapy. The CR2 for children with relapse age ≥10 years was lower than that for children aged 1-9.9 years. A positive fusion gene of ETV6-RUNX1 significantly increased the likelihood of achieving CR2 (P < 0.05). CR2 rates were 58.1%, 60.4%, and 85.7% for very early relapse, early relapse, and late relapse, respectively (P=0.043). In terms of relapse patterns, CR2 rates for patients with bone marrow relapse alone, extramedullary relapse alone, and combined bone marrow relapse were 57.9%, 87.5%, and 66.7%, respectively (P=0.036). Additionally, the CR2 for patients with B-ALL relapse was 68.8%, while that for T-ALL relapse was 50.0%, with no significant difference (**Table 5**).

**Table 4:** The relapse site and time of 146 relapse Children.

Variables	Very early relapse	Early relapse	Late relapse	No. total (n,%)
**Isolated bone marrow relapse**	42	27	23	92 (63.0%)
Isolated extramedullary relapse				
CNS relapse	14	8	2	24 (16.4%)
Testicular relapse	0	1	1	2 (1.4%)
Combined relapse*	6	10	12	28 (192%)
**No. Total (n, %)**	62 (42.5%)	46 (31.5%)	38 (26.0%)	146 (100%)

*Include bone marrow combined central, testicular, as well as lymph node and renal relapses.

**Table 5:** Comparison of CR, EFS, and OS in 105 relapsed children*.

Variables	No. of patients	CR2*(%)	P value	5-y event-free survival(%)	P value	5-y overall survival (%)	P value
Age at relapse							
1-9.9 year	76	72.4%	0.046	48.8%	0.011	64.8%	0.005
≥10 year	29	51.7%		22.6%		38.0%	
Sex							
Male	65	69.2%	0.480	46.1%	0.557	54.7%	0.485
Female	40	62.5%		33.1%		61.7%	
WBC count at diagnosis							
<100×10^9^/L	87	66.7%	1.000	41.5%	0.657	60.2%	0.057
≥100×10^9^/L	18	66.7%		42.9%		43.7%	
WBC count at relapse							
<50×10^9^/L	102	67.6%	0.257	43.0%	0.042	59.5%	0.008
≥50×10^9^/L	3	33.3%		0.0%		0.0%	
D46MRD at diagnosis							
<10^-4^	87	67.8%	0.583	43.1%	0.405	59.8%	0.196
≥10^-4^	18	61.1%		36.0%		46.3%	
MRD after relapse treatment							
<10^-4^	60	95.0%	<0.001	60.8%	<0.001	77.3%	<0.001
≥10^-4^	45	28.9%		16.0%		31.3%	
Fusion gene (positive)							
ETV6-RUNX1	12	91.7%	0.043	59.7%	0.137	76.4%	0.049
MLL-r	5	60.0%		40.0%		60.0%	
BCR-ABL1	5	40.0%		0.0%		0.0%	
TCF3-PBX1	11	36.4%		27.3%		36.4%	
Site of relapse							
Isolated bone marrow relapse	57	57.9%	0.036	21.1%	0.001	48.6%	0.082
Combined relapse	24	66.7%		50.8%		62.7%	
Isolated extramedullary relapse	24	87.5%		70.6%		73.8%	
Time of relapse							
Very early relapse	46	58.1%	0.043	28.4%	0.004	47.2%	0.011
Early relapse	31	60.9%		44.4%		55.3%	
Late relapse	28	85.7%		60.0%		77.0%	
Immunophenotyping							
B-ALL	93	68.8%	0.193	47.5%	<0.001	65.7%	<0.001
T-ALL	12	50.0%		0.0%		0.0%	

*EFS, event-free survival; OS, overall survival; CR2, second complete remission.

### Long-term survival after relapse

**Figure 1:**
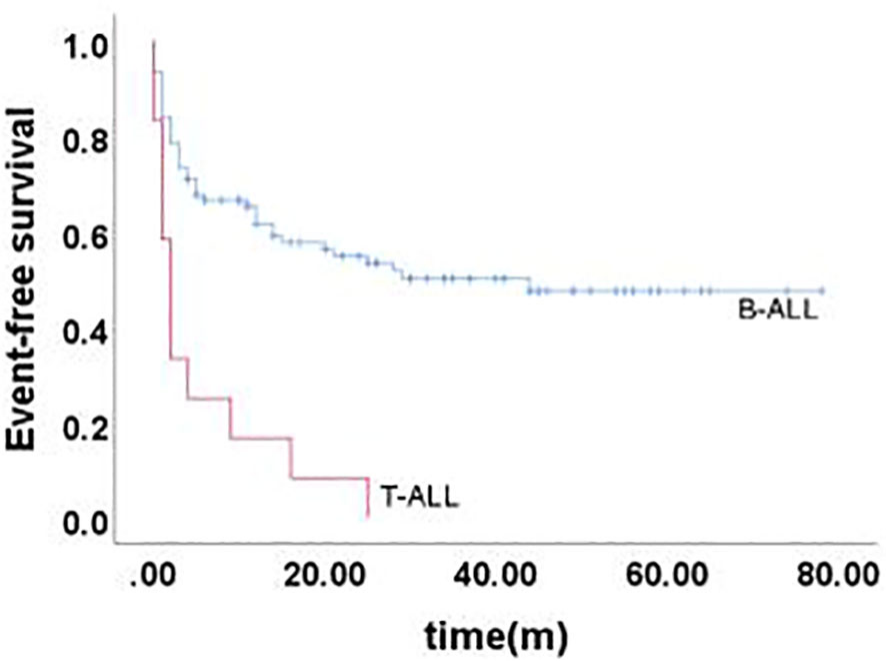
Event-free survival curves of children with different immunophenotypes, as assessed by Kaplan-Meier method.

**Figure 2:**
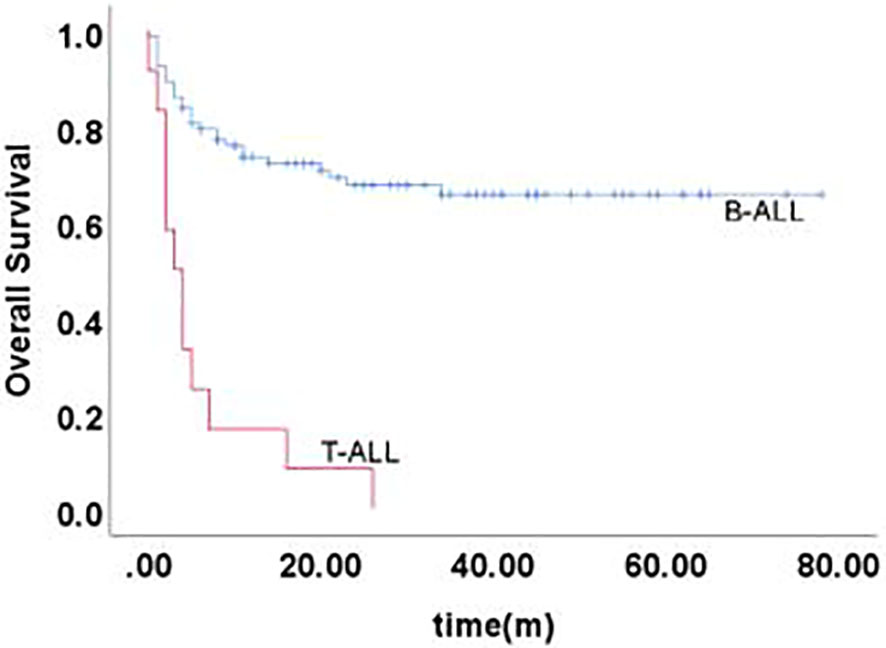
Overall survival curves of children with different immunophenotypes, as assessed by Kaplan-Meier method.

**Figure 3:**
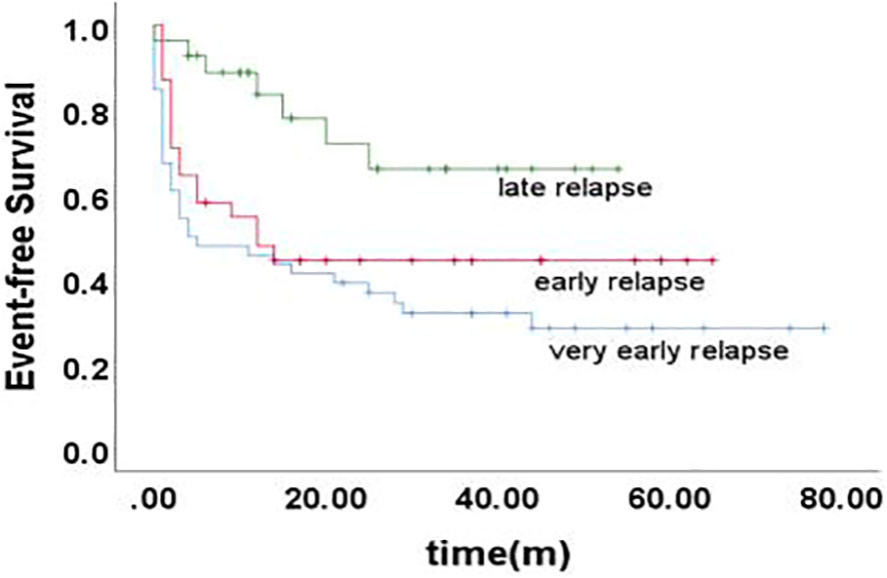
Event-free survival curves of children with different time of relapse, as assessed by Kaplan-Meier method.

**Figure 4:**
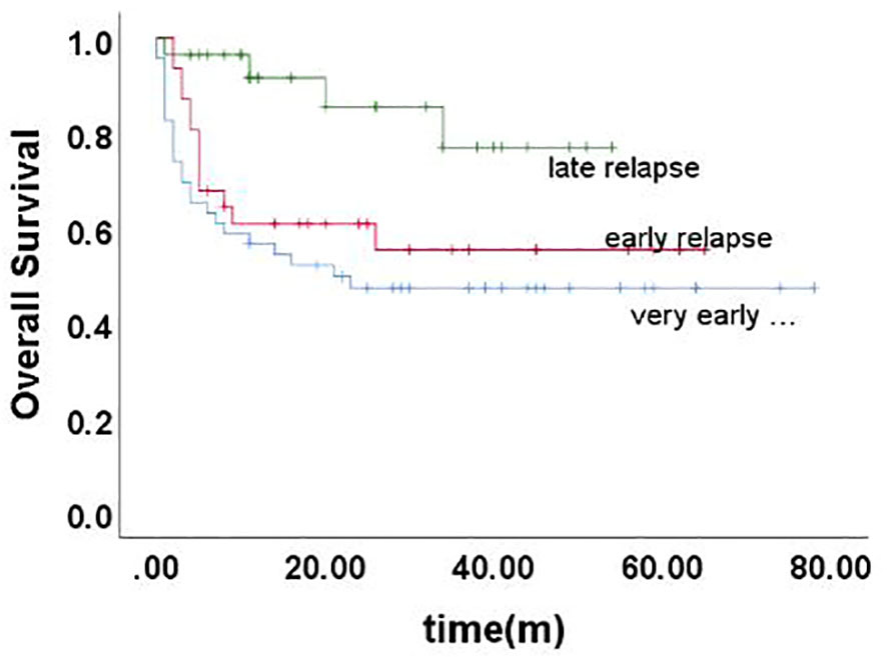
Overall survival curves of children with different time of relapse, as assessed by Kaplan-Meier method.

The 5-year EFS of 105 children was (41.6 ± 5.4)%, and the 5-year OS was (59.0 ± 5.3)%. The prognosis for T-ALL relapse was worse compared with B-ALL relapse, with 5-year EFS and OS being 47.5% vs 0.0% and 65.7% vs 0.0%, respectively (**Figures 1**, **2**). The 5-year EFS for children with very early relapse, early relapse, and late relapse was 28.4%, 44.4%, and 66.0%, respectively, and the 5-year OS was 47.2%, 55.3%, and 77.0%, respectively(**Figures 3**, **4**). The long-term survival rate of very early and early bone marrow relapses was only 38.1%, significantly lower than that of late bone marrow relapses (92.8%, P<0.05). The 5-year EFS and OS of bone marrow relapses were lower than those of isolated bone marrow relapse (P<0.05), but the difference was not statistically significant when compared with that of bone marrow-combined relapses. Two children with testicular relapses still had event-free survival up to the cutoff of the follow-up, while one case of early bone marrow combined testicular relapse died due to complications after CAR-T. Two children with late bone marrow combined testicular relapse experienced secondary relapse after treatment; one of them died after abandoning treatment for the second relapse after transplantation, and the other continued to receive CAR-T treatment outside the hospital. The 5-year EFS for relapses involving the center was 61.7%, and the 5-year OS was 68.3%. The 5-year EFS and OS after relapse were significantly higher in children with relapse age of 1-9.9 years than in children with relapse age ≥10 years, with rates of 48.8% vs 22.6%, P=0.011, and 64.8% vs 38%, P=0.005, respectively. The survival prognosis of MRD positive children after secondary induction therapy post-relapse was significantly lower than that of MRD negative children, with 5-year EFS and OS of 60.8% vs 16.0% and 77.3% vs 31.3%, respectively, demonstrating a significant difference (P < 0.001). Compared with fusion gene ETV6-RUNX1 negative patients, ETV6-RUNX1 positive relapsed patients exhibited notably higher 5-year EFS and OS, reaching 70.7% and 80.0%, respectively (P < 0.05). Conversely, the disparity in survival following relapse between individuals positive and negative for the fusion genes TCF3-PBX1, BCR-ABL1, and MLLr did not reach statistical significance. Among thirty children who underwent Hematopoietic Stem Cell Transplantation at our center, three experienced relapse post-transplantation, and tragically, one succumbed to encephalopathy following the procedure. Comparatively, transplanted children demonstrated a more favorable prognosis subsequent to relapse, showcasing a 5-year EFS and OS of 31.9% versus 66.2% and 46.8% versus 84.1%, respectively (P<0.01), when contrasted with non-transplanted patients.

### Factors affecting survival after relapse

Several factors influencing survival after relapse were subjected to analysis using a Cox proportional hazard model. These factors included the age at relapse, Minimal Residual Disease status following relapse treatment, White Blood Cell count at relapse, site of relapse, time to relapse, immunophenotype, the attainment of CR2, and the decision to undergo transplantation. The outcomes revealed that the site of relapse, immunophenotyping, the acquisition of CR2, and the decision for transplantation emerged as significant factors impacting the Event-Free Survival at 5 years post-relapse. Meanwhile, immunophenotype, the attainment of CR2, and the decision for transplantation were identified as significant factors influencing the Overall Survival at 5 years following relapse (**Table 6**).

**Table 6:** Multivariate analysis of survival among 105 relapsed children.

Variables	5-y event-free survival(%)			5-y overall survival (%)		
	P value	HR	95%CI	P value	HR	95%CI
**Age at relapse (vs 1-9.9y)**	0.400	1.29	0.71-2.32	0.158	1.62	0.83-3.15
**MRD after relapse treatment (vs <0.01%)**	0.933	0.96	0.39-2.36	0.504	1.44	0.49-4.20
**WBC count at relapse (vs <0.01%)**	0.123	2.97	0.74-11.90	0.194	2.90	0.58-14.50
**Site of relapse (vs Isolated bone marrow relapse)**	0.007	0.570	0.38-0.85	0.359	0.80	0.48-1.30
**Time of relapse (vs very early relapse)**	0.165	0.73	0.47-1.14	0.215	0.70	0.4-1.23
**Immunophenotyping (vs LR)**	0.001	3.60	1.71-7.60	0.003	3.18	1.50-6.77
**Get CR2 or not (vs no)**	<0.001	0.18	0.07-0.43	0.004	0.24	0.09-0.63
HSCT* or not (vs no)	0.012	0.38	0.18-0.80	0.009	0.23	0.08-0.69
**ETV6-RUNX1 (vs positive)**	0.967	1.03	0.28-3.70	0.666	3.18	1.49-6.77

*HSCT, hematopoietic stem cell transplantation.

## Discussion

After treating children ALL using the CCCG-ALL-2015 protocol in our center, we observed a 5-year CIR of 18.6%, the 5-year CIR for isolated CNS relapse was 2.8%, and the 5-year CIR for CNS relapse involvement was 4.6%.These findings contrast with those of Wenyu Yang et al., who reported a 5-year CIR of 1.1% for isolated central nervous system relapse and 1.7% for CNS involvement (6). The 146 children experienced relapses predominantly in the very early or early stages, with the most common site of relapse observed in the bone marrow. These findings align with reports from both domestic and international studies (7–9). There were 41 abandoned for various reasons. According to the family members, economic constraints (50.6%, p=0.0001) were the main reason for treatment abandonment, followed by the belief of incurability, severe side effects and concern over late complications (10). Regarding post-relapse treatment, the consensus continues to advocate for administering intensive chemotherapy, guided by risk stratification for relapsed children. Moreover, incorporating novel therapeutic approaches, such as targeted therapy, is recommended for high-risk cases. In our study, over 50% of relapsed ALL patients underwent re-treatment, achieving a secondary induction remission rate close to 70%, a result consistent with reported findings in China. Notably, age and time of recurrence emerged as factors influencing the acquisition of CR2. Additionally, akin to the initial diagnosis, the positive expression of the fusion gene ETV6-RUNX1 serves as a favorable prognostic indicator. Children with ETV6-RUNX1 positivity demonstrated a higher likelihood of attaining CR2, reinforcing the positive prognostic value associated with this genetic marker (11, 12).

It’s has lower 5-year EFS rate and OS rate after relapse compared to children with initial diagnosis in the country. According to Professor Shen Shuhong’s team, the 5-year EFS rate was 68.3 ± 1.4% and the OS rate was 80.0 ± 1.2% in patients with primary diagnosis. The 5-year EFS and OS rates following relapse among children with T-ALL in our center were notably lower than those observed in B-ALL. The mortality rate post-relapse in T-ALL was higher than that in B-ALL (66.7% vs 43.0%), consistent with reported findings (13, 14). This discrepancy may be attributed to T-ALL higher likelihood of experiencing induction failure compared to B-ALL. Additionally, T-ALL exhibited a higher proportion of very early and early relapses, along with a lower Minimal Residual Disease negative rate compared to B-ALL. An early recommendation based on our observations is the prompt initiation of allogeneic hematopoietic stem cell transplantation following T-ALL relapse (15). The time of relapse is an important prognostic factor whether it is a primary relapse or a post-transplantation re-relapse. In the present study, the 5-year OS post-very early relapse was notably lower at 47.2%, aligning with literature-reported results (16). A Turkish study similarly confirmed that the corresponding 3-year OS after transplantation for very early relapse and early relapse were 7.8% and 9.6%, respectively, *P*=0.041, which was significantly worse compared to late relapse (17). In our study, the 5-year EFS and OS for isolated bone marrow relapse were inferior to those for isolated extramedullary relapse, although not significantly reduced when compared to bone marrow-combined relapse. While historical studies confirmed a poorer prognosis for simple bone marrow relapse compared to bone marrow-combined relapse, recent research suggests comparable outcomes. Consequently, some studies have moved away from using isolated bone marrow relapse and bone marrow-combined relapse as the basis for risk stratification (18). Additionally, the 5-year EFS and OS rates of children aged 1-9.9 years at the time of relapse were significantly higher than those of children aged ≥10 years. This difference may be attributed to variations in the relapse stage within these age groups. Notably, the proportion of children aged ≥10 years experiencing relapse at the very early stage was markedly higher than that observed in children aged 1-9.9 years.

Furthermore, this study revealed that the prognosis of transplanted children following relapse was superior to that of non-transplanted counterparts. Independent research from a single center in China corroborated transplantation as a pivotal factor influencing the prognosis of relapsed ALL. The study recommended prompt transplantation for eligible families after achieving secondary induction remission. In cases where remission remains elusive, initiating specialized treatments, such as CAR-T therapy, as early as possible is advised to facilitate the transplant process (19, 20). In addition, minimal residual disease (MRD) after induction therapy becomes a key factor affecting survival in relapse and re-relapse after transplantation (17, 21).The survival prognosis of MRD-positive children following relapse-induced remission therapy was significantly lower compared to that of MRD-negative children, with 5-year EFS and OS rates of 60.8% vs 16.0% and 77.3% vs 31.3%, respectively(P<0.001). Therefore, standardized and individualized treatment approach, guided by risk stratification and incorporating innovative methods such as targeted therapy, is imperative to enhance the survival rates of relapsed children. For those with elevated MRD levels after reinduction, Hematopoietic Stem Cell Transplantation should be undertaken at the earliest opportunity post-remission (19, 22, 23).

The findings from this study, corroborated by both domestic and international research, has consistently indicated a grim prognosis for relapsed ALL. The survival rate in relapsed cases is significantly lower compared to primary ALL. Numerous factors contribute to this unfavorable outcome, encompassing the absence of standardized treatment protocols post-relapse, a heightened number of patients choosing to discontinue treatment, and increased resistance to chemotherapy, among other complexities. Specifically, the prognosis is even more discouraging for certain subgroups: Children with relapsed T-cell ALL, those who experience relapse after the age of 10, and those with very early or early relapses face a particularly bleak outlook. Furthermore, those unable to attain CR2 status and forgo hematopoietic stem cell transplantation after relapse exhibit a significantly poorer prognosis.

Addressing these challenges demands the implementation of risk-stratified treatment approaches tailored for relapsed children. This necessitates a nuanced comprehension of the criteria guiding hematopoietic stem cell transplantation. Moreover, continuous exploration and development of novel treatment modalities remain pivotal to augment survival rates within this particular cohort of children following relapse. Through the adoption of these strategies, a concerted effort is underway to enhance the overall prognosis and outcomes for children contending with relapsed ALL.

## Data Availability

The original contributions presented in the study are included in the article/supplementary material. Further inquiries can be directed to the corresponding author.
